# Effect of anthropogenic sulphate aerosol in China on the drought in the western-to-central US

**DOI:** 10.1038/srep14305

**Published:** 2015-09-22

**Authors:** Sang-Wook Yeh, Rokjin J. Park, Minjoong J. Kim, Jaein I. Jeong, Chang-Keun Song

**Affiliations:** 1 Department of Marine Sciences and Convergent technology, Hanyang University, Ansan, Korea; 2 School of Earth and Environmental Sciences, Seoul National University, Seoul, Korea; 3 Department of Climate and Air Quality, National Institute of Environmental Research, Incheon, Korea

## Abstract

In recent decades, droughts have occurred in the western-to-central United States (US), significantly affecting food production, water supplies, ecosystem health, and the propagation of vector-borne diseases. Previous studies have suggested natural sea surface temperature (SST) forcing in the Pacific as the main driver of precipitation deficits in the US. Here, we show that the aerosol forcing in China, which has been known to alter the regional hydrological cycle in East Asia, may also contribute to reducing the precipitation in the western-to-central US through atmospheric teleconnections across the Pacific. Our model experiments show some indications that both the SST forcing and the increase in regional sulphate forcing in China play a similar role in modulating the western-to-central US precipitation, especially its long-term variation. This result indicates that regional air quality regulations in China have important implications for hydrological cycles in East Asia, as well as in the US.

The western-to-central United States (US) has experienced anomalously low precipitation over the past decades (Supplementary Fig. S1A), and this drought has significantly affected its economy^1^. Previous studies have suggested that the natural sea surface temperature (SST) forcing in the Pacific, such as La Niña-like cooling and the Pacific Decadal Oscillation, might be the cause of the precipitation deficit in the US^2^,^3^,^4^. In addition, other results showed that wet or dry decades in the US were associated with changes in the atmospheric circulation across the Pacific, which were also connected to the SST pattern in the global ocean^5^. Furthermore, a recurrent teleconnection pattern of circulation anomalies between East Asia and North America^6^,^7^,^8^ implies the existence of other factors from East Asia that lead to persistent drought in the US. In this study, we pay particular attention to the increase of regional aerosol forcing in East Asia over the past decades, which is mainly driven by the increase of anthropogenic emissions in China that is experiencing the highest growth rate since 2000^9^.

Atmospheric aerosols are known to affect regional synoptic meteorology by directly absorbing and scattering solar radiation, a phenomenon referred to as aerosol forcing. The aerosol forcing increase has modified the regional hydrological cycle, such as by enhancing or reducing precipitation in China during certain seasons^10^,^11^,^12^,^13^. Subsequently, it is likely that the changes in precipitation and circulation in China may have some impact on the downwind regions via modulation of the atmospheric circulation. In this study, we explore this possible relationship, focusing particularly on the change in long-term mean precipitation in China and the western-to-central US (120°W–90°W, 30°N–40°N) before and after the aerosol forcing in China was significantly increased.

We use the monthly Climate Prediction Center Merged Analysis of Precipitation (CMAP)^14^ and National Centers for Environmental Prediction (NCEP)/Department of Energy (DOE) reanalysis II (RA2)^15^ datasets to examine the observed global circulation pattern change and precipitation trends. We also use an atmospheric general circulation model (AGCM) and sulphate aerosol concentrations from a global 3-D atmospheric chemistry model (GEOS-Chem) driven by the assimilated meteorology from the Modern-Era Retrospective Analysis for Research and Applications, National Aeronautics and Space Administration (MERRA NASA)^16^ to explore the relative contributions of natural SST forcing and anthropogenic aerosol forcing. We focus our observational analysis on the period 1985–2010, which is limited by the reliability of precipitation observations and by the availability of anthropogenic emissions used in the model.

Figure 1 shows the regression patterns of the NCEP RA2 geopotential height anomaly at 500 hPa and wind anomaly at 850 hPa in the Northern Hemisphere, and of the CMAP precipitation anomaly in the US. Both are obtained by performing a regression against the time series of the observed precipitation in southeastern China (105°E–120°E, 20°N–35°N) for 1985–2010. Note that we remove the trends in all variables in Fig. 1 before calculating the regression coefficients. The regressed geopotential height anomalies show a zonally elongated atmospheric teleconnection pattern linking the variability of summertime precipitation over southeastern China and the western-to-central US^5^,^6^ (Fig. 1A). It is found that the anomalous high geopotential height is observed in the western-to-central US. Note that a similar result is shown in the MERRA reanalysis dataset (Supplementary Fig. S2). This circulation pattern is likely associated with the below-normal precipitation in the western-to-central US, as shown in Fig. 1B. Therefore, the above-normal precipitation in southeastern China is associated with the below-normal precipitation in the western-to-central US through the atmospheric teleconnections across the Pacific on interannual timescales, without a trend. Note that the simultaneous correlation coefficient between the precipitation variability in southeastern China and the western-to-central US (120°W–90°W, 30°N–40°N) is –0.43, which is statistically significant at the 90% confidence level.

In addition, the total precipitation variability from the CMAP observations shows an increasing trend of summer precipitation in southeastern China for 1985–2010 (Fig. 2A). Although the linear precipitation trend in southeastern China is barely significant at the 80% level, an increasing (decreasing) precipitation in summer rainfall is evident over southeastern (northeastern) China from 1985–1994 to 2001–2010 (Fig. 2B). Furthermore, a negative trend of precipitation in the western-to-central US for 1985–2010 is also found (Supplementary Fig. S1B). Therefore, we think that the decrease of precipitation in the western-to-central US is related to the increase of precipitation in southeastern China for long-term periods. In other words, a more frequent occurrence of above-normal precipitation in southeastern China (Fig. 2C) is associated with a more frequent occurrence of below-normal precipitation in the western-to-central US (Fig. 1B), resulting in the decrease of mean precipitation in the western-to-central US in recent decades. Previous studies suggested that the increase in rainfall amount in southeastern China in the past decade could be associated with the SST forcing over the tropical Pacific^17^,^18^. We further explore a possible role of the anthropogenic sulphate aerosol forcing that significantly differs in mid- and southeastern China for 2001–2010 (see Fig. 3D).

Figure 3A shows the simulated mean distribution of the sulphate aerosol column burdens, which are proxies for the anthropogenic emissions from the surface to 1.4 km for 1985–2010. The sulphate aerosols are mostly found in the low-level troposphere; therefore, the change in integrated height from the surface has little effect on the present results. The sulphate aerosol concentrations are high in the continental mid-latitude regions of the Northern Hemisphere. The highest values are found in the central-eastern US, central Europe, and eastern China, and a similar spatial distribution is found in the average sulphate burdens during the boreal summer (June–July–August) (Fig. 3B). However, the long-term trends of the summertime sulphate burdens differ. While they have gradually decreased in the central-eastern US (90°W–70°W, 30°N–45°N) and central Europe (10°E–30°E, 40°N–55°N), the sulphate burdens have increased in southeastern China in 1985–2010 (Fig. 3C,D). In particular, China shows a sharp increase after the early 2000s (Fig. 3D) because of the enhanced fossil fuel use during the past decade^14^. This dramatic increase of aerosol forcing in China has affected the temperature of the land surface, along with regional vertical motions and atmospheric circulation,^10^,^11^ and could induce a precipitation change in southeastern China (see also Supplementary Figs S3 and S4).

To further explore the influence of the increase of sulphate aerosol and SST forcing on the observed precipitation variability in southeastern China, we conduct three sets of ensemble model experiments using Community Atmosphere Model version 5.1.1, which consists of the atmospheric component in the Community Earth System Model (http://www.cesm.ucar.edu/models/cesm1.0/cam/). The first set includes model simulations prescribed with the historical SST over the globe without the Asian SO_2_ emission for 1985–2010 (hereafter referred to as the SST-run). The second set includes model runs prescribed with the climatological monthly mean SST averaged for 1985–2010, but with the time-varying SO_2_ emission in Asia (hereafter referred to as the SO_2_-run). Finally, we use the historical SST for 1985–2010 and the time-varying SO_2_ emission in Asia in model runs (hereafter referred to as the SST-SO_2_ run). Each set of experiments is performed with four ensemble members, the ensemble mean of which is presented in this study. A detailed explanation of the model runs and some simulations of the atmospheric circulation and its associated precipitation variability during the summer in the SST-SO_2_ run is provided in the Supplementary Information (Supplementary Figs S5, S6, and S7). By directly comparing the results from the three sets, we are able to identify the influences of the SST forcing and sulphate aerosol forcing in China on the precipitation changes in southeastern China and the western-to-central US.

Figures 4A,B display the difference of summer precipitation simulated in the SST-run and the SO_2_-run between the two periods (i.e., 2001–2010 minus 1985–94), respectively. It is evident that the precipitation amounts in both the SST-run (Fig. 4A) and the SO_2_-run (Fig. 4B) have increased in southeastern China, although detailed structures differ compared to the observations (see Fig. 2B). For example, the observed increase shows a wider latitudinal distribution than both simulations. In spite of that, the model results generally indicate that both the SST forcing and sulphate aerosol forcing are responsible for the precipitation changes in southeastern China between the two periods (see also Supplementary Fig. S8). In particular, we emphasize that the increased sulphate aerosol contributes to increase the amount of precipitation in southeastern China from 1985–94 to 2001–2010. That is, the increase of aerosol forcing in southeastern China has affected the temperature gradient in the meridional direction in the SO_2_-run (Supplementary Fig. S9A). With dynamical processes similar to those shown in the observations, the aerosol-induced temperature change leads to secondary circulation, accompanied by jet stream weakening and rising motion over southeastern China (Supplementary Fig. S9B), resulting in increased precipitation.

The time series of anomalous precipitation average in southeastern China during the summer also shows a pronounced change before the mid-1990 s and after the early 2000 s in the SO2-run (Fig. 4C). The above-normal precipitation frequently occurs in southeastern China after the early 2000 s, which is reminiscent of the observations (Fig. 2C). Concurrently, the precipitation changes in southeastern China are connected to those over North America via the atmospheric teleconnections in the SO_2_-run. Supplementary Fig. S10A shows the regression pattern of the geopotential height anomalies at 500 hPa in the Northern Hemisphere against the precipitation variability in southeastern China in the SO_2_-run. The structure of atmospheric teleconnection in the SO_2_-run is different compared to the observations (Fig. 1A), which might be due to the model’s inability to correctly simulate the atmospheric circulation associated with the precipitation variability. Such model deficiencies in simulating the precipitation variability and the associated atmospheric circulation are also found in most of the CMIP3 and CMIP5 models^19^. In spite of this, we argue that a wave train signal across the Pacific is associated with atmospheric teleconnections from the precipitation over southeastern China. An anomalous high geopotential height over the western-to-central US is found, which is probably associated with the precipitation decrease in the western-to-central US. Therefore, more frequent occurrence of above-normal precipitation in southeastern China after the early 2000 s in the SO_2_-run is associated with more frequent occurrence of below-normal precipitation in the western-to-central US (Fig. 4D). This leads to the reduction of mean precipitation in the same region in the early 2000 s (see also Fig. 5C).

We find a similar response in the SST-run that reproduces the observed decrease of rainfall amount over the US (Fig. 5B), which is consistent with previous studies, to some extent^2^,^3^,^4^,^20^,^21^,^22^ (Supplementary Fig. S11). Anomalous high geopotential height over the western-to-central US, which is associated with atmospheric teleconnections from the precipitation variability over southeastern China, is found (Supplementary Fig. S10B). There are some similarities and differences with the SO_2_-run, which is indicative of different roles of SST forcing and regional aerosol forcing (see Supplementary Information). Note that the changes of precipitation amount averaged in the western-to-central US between the two periods (2001–2010 minus 1985–1994) are −0.13 mm/day, −0.15 mm/day, and −0.12 mm/day in the observation, SST-run, and SO_2_ run, respectively. Therefore, we conclude that an increase of sulphate aerosol forcing in China may contribute to modulate the hydrological cycle in the western-to-central US by perturbing atmospheric teleconnections across Pacific, which is comparable to the SST forcing. This result bears the important implication that a regional change of anthropogenic aerosol forcing is capable of modulating the hydrological cycle over regions far from the aerosol source. However, it should be also noted that there is a possibility that the aerosols themselves are advected downstream, and may impact SST forcing, resulting in changes in atmospheric circulation and precipitation. Because the boundary SST forcing is prescribed in all the experiments, such impacts are not considered in the present study.

## Methods

### Model data

Effects of sulphate aerosol forcing in China are diagnosed from the simulation of the global chemical transport model (GEOS-Chem), NCAR CAM5 (Community Atmosphere Model version 5) model, and observations. The sulphate aerosol concentrations are obtained from the GEOS-Chem simulation for the period 1985–2010, using the assimilated meteorological data from MERRA NASA. GEOS-Chem has been extensively evaluated for aerosol simulations everywhere, including Asia^23^, where it reproduces realistic sulphate aerosol forcings, which vary on seasonal to interannual timescales along with the trends. To examine the influence of an increase of sulphate aerosol over China, we use NCAR CAM5 to conduct the three runs: the SST-run with the observed SST and no Asian SO_2_ emission, the SO_2_-run using yearly varying SO_2_ emissions with climatological SST, and the SST-SO_2_ run with observed SST along with varying SO_2_ emissions in Asia. Each model run produces four different ensembles by changing the initial conditions. Therefore, we conduct a total of 12 model runs for 1985–2010. The set of simulations begins on January 1, 1985, with the same initial meteorological conditions, obtained from the 10-year CAM5 spin-up integration from 1975. We conduct four CAM5 ensemble simulations for each experiment, wherein each member of the ensembles begins on different dates, such as January 1–4, 1985.

### Statistical analysis

Linear regression and composite analysis are used to identify the teleconnective effects of sulphate aerosol forcings in the observation data and model outputs.

### Seasonality

All calculations in this paper are performed for the boreal summer season (June–July–August).

### Graphics software

All maps and plots were produced using Grid Analysis and Display System (GrADS, http://cola.iges.org).

## Additional Information

**How to cite this article**: Yeh, S.-W. *et al.* Effect of anthropogenic sulphate aerosol in China on the drought in the western-to-central US. *Sci. Rep.* **5**, 14305; doi: 10.1038/srep14305 (2015).

## Supplementary Material

Supplementary Information

## Figures and Tables

**Figure 1:**
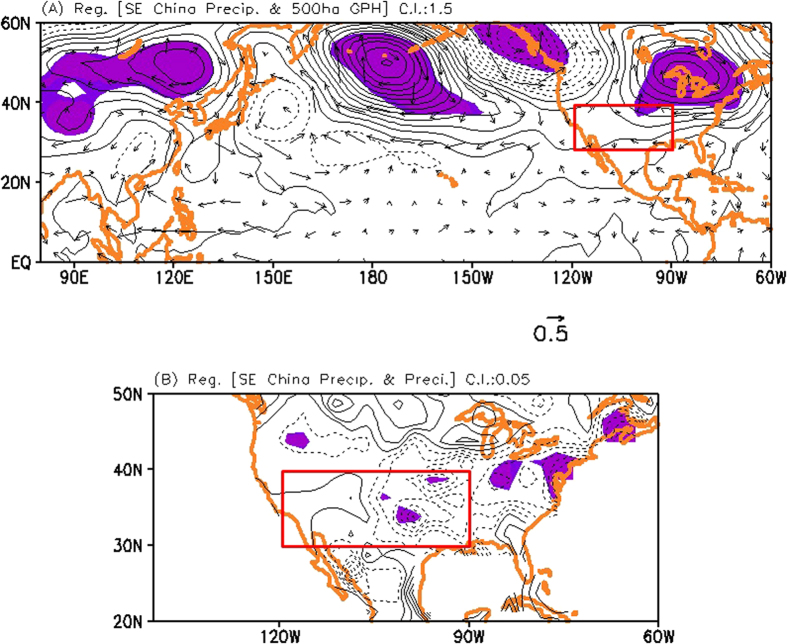
(**A**) Regressed geopotential height anomalies at 500 hPa from NCEP/DOE RA2 against the time series of observed precipitation averaged over southeastern China (105°E–120°E, 20°N–35°N). Contour interval is 1.5 m/mm/day. Vectors indicate the regressed winds at 850 hPa. (**B**) Same as (**A**), but for the regressed precipitation anomalies in the United States, and the unit in (**B**) is non-dimensional. Contour interval is 0.05. Shading in (**A**) and (**B**) denotes the statistical significance above the 90% confidence level. Grid Analysis and Display System (GrADS, http://cola.iges.org) was used to create the maps and plots in Fig. 1.

**Figure 2:**
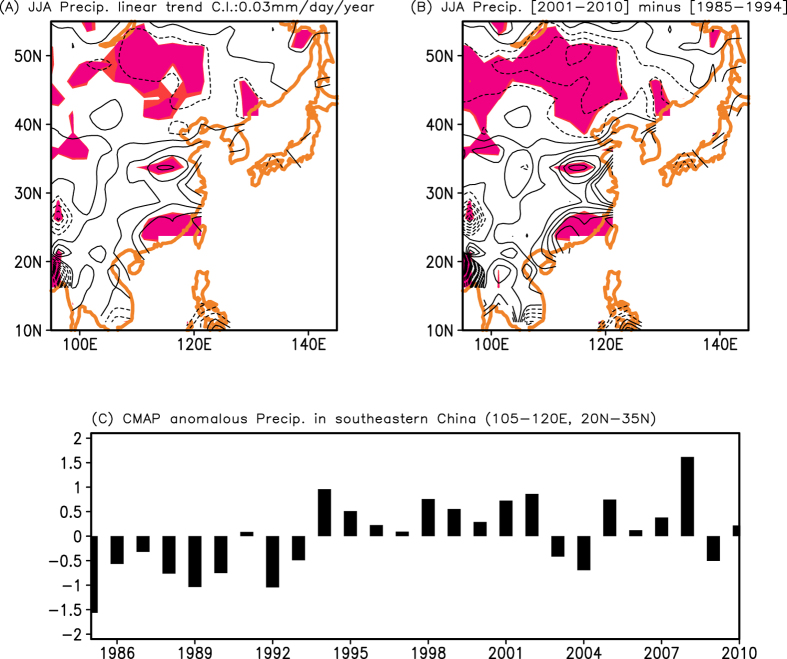
(**A**) Linear trend of CMAP observation for 1985–2010. Shading denotes the statistical significance at the 80% confidence level. Contour interval is 0.03 mm/day/year. (**B**) Difference in summer precipitation from 1985–1994 to 2001–2010. Shading denotes the statistical significance at the 90% confidence level. (**C**) Time series of anomalous precipitation averaged in southeastern China (105°E–120°E, 20°N–35°N) during the summer in the CMAP dataset. Units in Fig. 2B,C are mm/day. GrADS (http://cola.iges.org) was used to create the maps and plot in Fig. 2.

**Figure 3:**
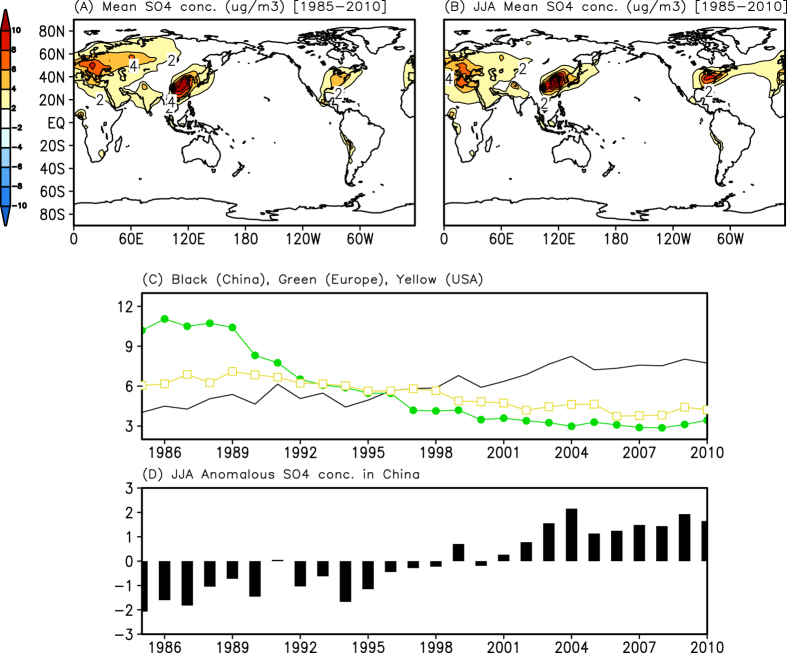
Distribution of sulphate aerosol simulated by GEOS-Chem. (**A**) Mean distribution of sulphate aerosol column burdens simulated by GEOS-Chem from the surface to a height of 1.4 km for 1985–2010. (**B**) Same as (**A**), but for boreal summer (June–July–August). (**C**) Time series of sulphate aerosol column (approximately 1.4 km) averaged in southeastern China (105°E–120°E, 20°N–35°N, black line), central Europe (45°E–55°E, 10°N–30°N, green), and the central-eastern United States (90°W–110°W, 35°N–45°N, yellow) during the summer for 1985–2010. (**D**) Time series of anomalous sulphate aerosol averaged in mid- and southeastern China during the summer for 1985–2010. GrADS (http://cola.iges.org) was used to create the maps and plots in Fig. 3.

**Figure 4:**
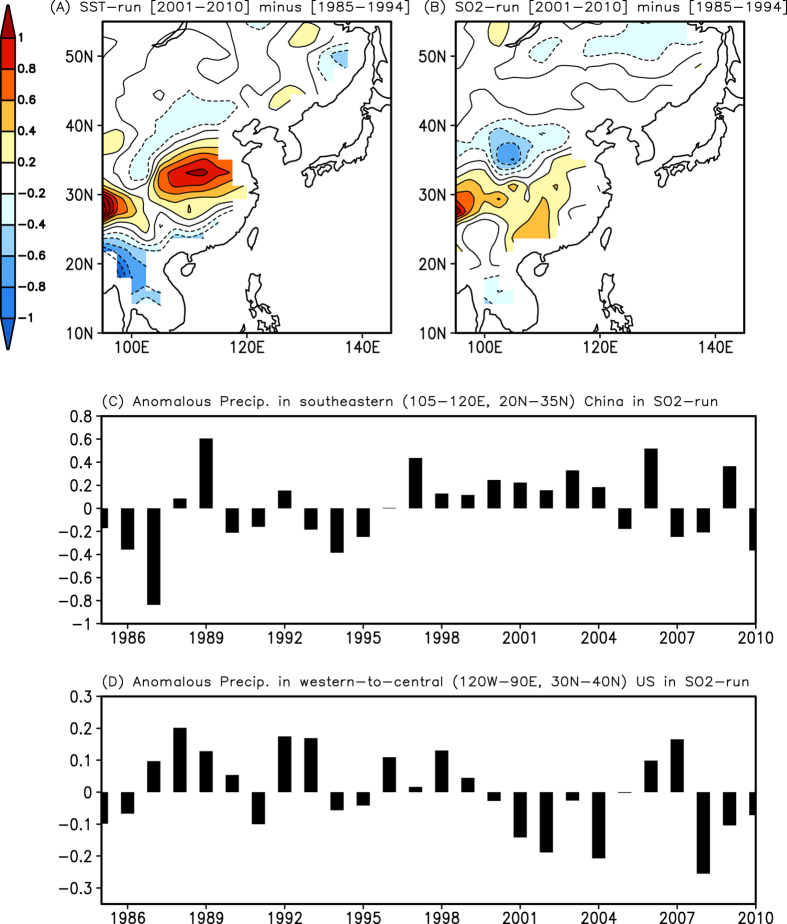
(**A**) Difference of summer precipitation simulated in the SST-run between the two periods (i.e., 2001–2010 minus 1985–1994). Contour interval is 0.2 mm/day/year. (**B**) Same as (**A**), but for the SO_2_-run. (**C**) Time series of anomalous precipitation averaged in southeastern China during the summer in the SO_2_-run. Unit is mm/day/year. (**D**) Fig. 4D is the same as Fig. 4C, but for anomalous precipitation averaged in the western-to-central US (120°W–90°E, 30°N–40°N). GrADS (http://cola.iges.org) was used to create the maps and plots in Fig. 4.

**Figure 5:**
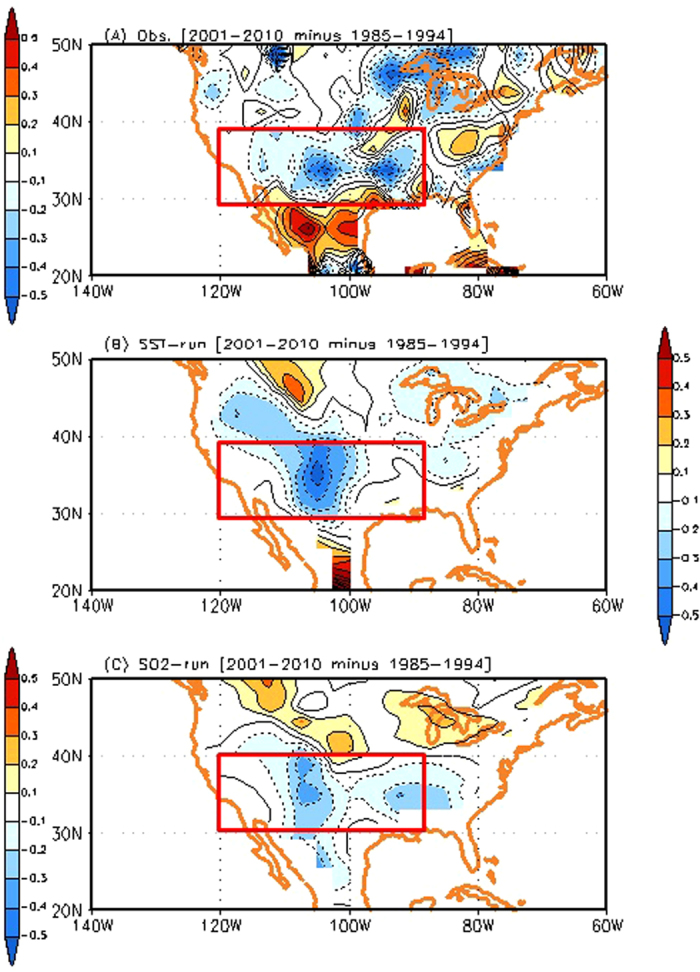
(**A**) Difference of precipitation in the US between 1985–1994 and 2001–2010 during the summer. Difference in CMAP dataset between 1985–1994 and 2001–2010 during the summer (2001–2010 minus 1985–1994). (**B**), (**C**) Same as (**A**) except for the SST-run the SO_2_-run, respectively. Units in Fig. 5A–C are mm/day. GrADS (http://cola.iges.org) was used to create the maps in Fig. 5.
