# Supplementary material for: Effect of anthropogenic sulphate aerosol in China on the drought in the western-to-central US
Source: Sci Rep. 2015 Sep 22;5:14305. doi: 10.1038/srep14305 (PMC4585750; doi:10.1038/srep14305)
Supplement: Supplementary Information [file srep14305-s1.doc]

Effect of anthropogenic sulphate aerosol in China

on the drought in the western-to-central US

**Authors:** Sang-Wook Yeh^1^, Rokjin J. Park^2*^, Minjoong J. Kim^2^, Jaein I. Jeong^2^, Chang-Keun Song^3^

**Affiliations:**

^1^ Department of Marine Sciences and Convergent technology, Hanyang University, Ansan, Korea.

^2^ School of Earth and Environmental Sciences, Seoul National University, Seoul, Korea.

^3^Department of Climate and Air Quality, National Institute of Environmental Research, Incheon, Korea.

*Correspondence to: [rjpark@snu.ac.kr](mailto:rjpark@snu.ac.kr)

**Supplementary Information**

**GEOS-Chem model description and simulations.**

We use the global chemical transport model (GEOS-Chem) to conduct a fully coupled oxidant-aerosol simulation . The GEOS-Chem model v9-01-02 (http://www.as.harvard.edu/chemistry/trop/geos) uses the assimilated meteorology from the Modern-Era Retrospective Analysis for Research and Applications National Aeronautics and Space Administration (MERRA NASA) . The data include winds, convective mass fluxes, temperature, clouds, and precipitation at a 3-h frequency with a horizontal resolution of 0.5° × 0.666° and 72 hybrid pressure-sigma levels extending up to 0.01 hPa. We degrade these meteorological fields to a horizontal resolution of 2° × 2.5° and 47 vertical levels for computational expediency.

GEOS-Chem includes more than 80 species and 300 reactions for detailed O_3_-NO_X_-hydrocarbon chemistry coupled with aerosol chemistry. The aerosol simulation includes H_2_SO_4_-HNO_3_-NH_3_ aerosol thermodynamics , and the formation of these aerosols is computed locally with the ISORROPIA II aerosol thermodynamic equilibrium model . The model uses national emission inventories for anthropogenic species where available, including the European Monitoring and Evaluation Program (EMEP) inventory for Europe, the EPA 1999 National Emissions Inventory (NEI99, U.S. Environmental Protection Agency) for the U.S., and otherwise default values from the Global Emission Inventory Activity (GEIA) scaled to the simulation years . We use the anthropogenic emissions from the gridded inventory for 2000 over the Asian domain (60°E–158°E and 13°S–54°N) only . The Asian emissions of NO_X_, SO_2_, and NH_3_ are 9.1 Tg N y^–1^, 18.9 Tg S y^–1^, and 21.8 Tg N y^–1^, respectively. We subsequently apply the annual scale factors of the Regional Emission inventory in Asia (REAS) for 1985-2010 to the Streets et al. emissions to impose interannual variations in the model. The REAS inventory includes anthropogenic emissions for 1985-2003 estimated based on the fuel combustion and industrial sources and projected emissions after 2004. GEOS-Chem simulations have been applied to a number of air chemistry issues, including important tropospheric aerosols and they have been extensively evaluated by comparison against observations in the U.S., Europe, and Asia. The long-term simulations of sulphate aerosol concentrations exhibit a good agreement with the observations at the EANET sites in Asia ^23^.

**Relationship of sulphate aerosol, precipitation and atmospheric teleconnections**

Supplementary Fig. S1A indicates the time series of anomalous precipitation averaged in the US (120°W-90°W, 30°N-40°N) during the boreal summer (June-July-August) for the period of 1985-2010 using the CMAP precipitation datasets. Supplementary Fig. S2 is the same as in Fig. 1A in the main text except the Modern-Era Retrospective Analysis for Research and Applications National Aeronautics and Space Administration (MERRA NASA). Similar to Fig. 1A, the regressed geopotential height anomaly shows a zonally elongated atmospheric teleconnection pattern linking the variability of summertime precipitation over southeastern China and the western-to-central US. The anomalous high geopotential over the western-to-central US is evident, which is likely associated with the precipitation decrease in the same region.

Supplementary Fig. S3A indicates the differences of zonal (105°E-120°E) averaged temperature between the two periods (2001-2010 minus 1985-1994) using the NCEP RA2. It is evident that there exists a strong gradient of the zonal temperature in the meridional direction. That is, the zonal temperature in northeastern China is much warmer in the low-level troposphere than that in southeastern China, which is mainly associated with the radiative cooling effect from the enhancement of sulphate aerosol in southeastern China. This leads the weakening in the meridional temperature gradient for 2001-2010 compared to for 1985-1994. In addition, this is also associated with decelerating upper level jet stream at the jet exit region, as indicated by the strong negative upper level zonal wind anomaly at 35–45°N (Supplementary Fig. S3B). Subsequently, the secondary circulation induced by jet stream weakening, with rising motion around 20°N~35°N and sinking motion around 45°N, which is consistent with the convergence and divergence field at upper levels (Supplementary Fig. 3C). These secondary circulations are consistent with the precipitation difference in Fig. 2A in the main text. Note that similar results are obtained from using a different reanalysis dataset (not shown here).

Supplementary Fig. S4 indicates the regressed precipitation against with the time series of sulphate aerosol averaged in the mid- and southeastern China (105°E-120°E, 20°N-35°N). Simple regression analysis indicates that the increase of sulphate aerosol in the eastern China is associated with the increase (decrease) of precipitation amount in southeastern (northeastern) China.

Supplementary Fig. S8 displays the difference of summer precipitation simulated in the SST-SO_2_ run between the two periods (i.e., 2001-2010 minus 1985-94). Unlike the observation, the SST-run and SO_2_-run (Fig. 2 and Fig. 4 in the main text), the increase of precipitation amount in southeastern China is not significant along with detailed structures. The increase of precipitation amount is very limited in the southeastern China model, which is somewhat different from that in both the SST-run and SO_2_-run. This may indicate that the contribution of the SST forcing and the regional sulphate aerosol forcing is not linear to increase the precipitation amount in the southeastern China, which should be explored more details. Supplementary Fig. S10B displays the regressed geopotential height anomalies at 500 hPa in the Northern Hemisphere against with the precipitation variability in southeastern China in the SST-run. The structure of atmospheric teleconnection in the SST-run is different compared to the SO_2_-run (Supplementary Fig. S10A). A wave-train signal associated with the precipitation variability in southeastern China is originated from the southeastern China in the SO_2_-run, which is similar to the observation in spite of some discrepancies in detailed structures. In contrast, that in the SST-run is originated from the western tropical Pacific, which is different from the observation. However, the pattern correlation coefficient between the regressed geopotential heights in the observation (Fig. 1A) and the SO_2_-run in the entire domain (0-60°N, 80°E-60°W) (i.e., 0.23) is not different from that between the observation and the SST-run (Supplementary Fig. S10B) (i.e., 0.23).

We emphasize the anomalous high geopotential height over the western-to-central US in both runs, which is likely associated with the precipitation decrease. The simultaneous correlation coefficient between the precipitation and the geopotential height at 500hPa in the western-to-central US is -0.66, -0.49 and -0.29 in the observation, the SO_2_-run and the SST-run, respectively. This indicates that the anomalous high geopotential height at 500hPa is associated with the reduction of precipitation in the western-to-central US. Note that the correlation coefficients in the observation and the SO_2_-run are statistically significant at the 95% confidence level and that in the SST-run is statistically significant at the 85% confidence level. This may indicate that the atmospheric teleconnections associated with both the SST forcing and the regional aerosol forcing are able to modulate the anomalous circulation over the western-to-central US across the Pacific. In other words, the regional aerosol forcing without the SST forcing can lead the changes in atmospheric circulation across the Pacific comparable to the SST forcing only.

**Community Atmosphere Model simulations**.

We performed model simulations using the NCAR CAM5 (Community Atmosphere Model version 5) model coupled to the Community Land Surface Model version 4 ^30^. The CAM5 model is based on the finite volume (FV) dynamical core at a 1.9°×2.5° horizontal resolution and with 30 vertical levels. For this study, the ocean and ice modules were not fully coupled but were communicated to the atmosphere via an oceanic surface boundary condition, given as mid-month values of sea surface temperature, as well as sea ice fractions over the polar region. The sea surface temperature and the sea ice fractions are time series data constructed by concatenating and interpolating global HadISST data from the Met Office Hadley center ^31^ to the FV core grids of CAM5. The observed multi-year SST forcing may also serve the purpose of eliminating the uncertainty related to interactions between the atmosphere and the ocean. For aerosol simulations, the CAM5 uses a three-mode version of the modal aerosol model (MAM3), which resolves three different modes: the Aitken, accumulation and coarse modes . The aerosol species in MAM3 include sulphate, primary organic matter, secondary organic matter, black carbon, soil dust, and sea salt aerosols. The internal mixture between species is considered in the MAM3. For sulphate aerosol simulations, simple gas-phase chemistry is used, including DMS oxidation by OH to form SO_2_, SO_2_ oxidation by OH to form H_2_SO_4_, H_2_O_2_ production, and H_2_O_2_ loss. The total number of transported aerosol tracers in MAM3 is 15. The transported gas species include SO_2_, H_2_O_2_, DMS, H_2_SO_4_, and a lumped, semi-volatile organic species. Oxidant concentrations (O_3_, OH, and HO_2_) are interpolated from monthly averages taken from simulations by a chemistry-climate model (CAM-Chem) . A detailed description of MAM3 can be found in the literature .

In this study, we focus on changes in sulphate aerosols in China and their effects on meteorology. For this purpose, we updated Asian anthropogenic SO_2_ emissions in CAM5 with the gridded inventory for 2000 over the Asian domain (60°E–158°E and 13°S–54°N) ^27^. The Asian emission of SO_2_ for 2000 is 18.9 Tg S y^–1^. Next, we apply annual scale factors of the Regional Emission inventory in Asia (REAS) for 1985-2010 to the Streets et al. emissions to impose interannual variations in the model. Other aerosols were simulated by the MAM3 in CAM5 . Supplementary Fig. S5 displays the time series of the concentration of simulated SO_4_^2-^ in the SO_2_-run. As in Fig. 3D in the main text, the sulphate aerosol concentrations in China are distinct before the mid-1990s and after the early-2000s.

To examine the performance of the CAM5 to simulate the atmospheric circulation and the associated precipitation variability in East Asia monsoon during the boreal summer (June-July-August), we first define the East Asian summer monsoon (EASM) index^35^, which is used by the NOAA Climate Prediction Center (<http://www.cpc.ncep.noaa.gov/products/Global_Monsoons/Asian_Monsoons/monsoon_index.shtml>) as follows.


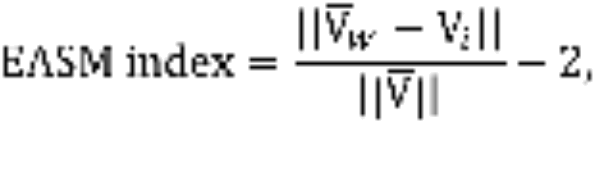


where 
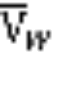
 and 
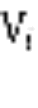
 are the reference climatological winter wind vector and monthly wind vector at point
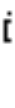
, respectively, and 
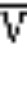
 = (
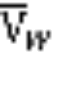
 + 
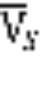
)/2 is the climatological mean wind vector. In this study, 
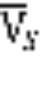
 is the climatological summer wind (for the Northern Hemisphere, taking 
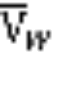
 = 
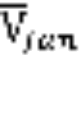
 and 
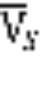
 = 
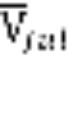
). The norm ||A|| is defined as follows:


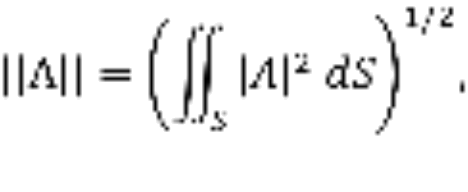


where S denotes the domain of integration. The EASM index well represents the atmospheric circulation associated with the rainfall variability in central-to-southern China during summer.

It is found that the spatial pattern in the precipitation variability associated with the EASM index in the SST-SO_2_ run is not much different from the observation (see Supplementary Fig. S6). To examine the variability in the EASM, we calculate the EASM index in the observation and the SST-SO_2_ run for 1985-2010 (Supplementary Fig. S7). It is found that the variability of EASM index is reasonably simulated in the SST-SO_2_ run in comparable to the observation. Note that a simultaneous correlation coefficient between the two time series is 0.52, which is statistically significant at the 95% confidence level.

24. Bey, I. *et al.* Global modeling of tropospheric chemistry with assimilated meteorology: Model description and evaluation. *J. Geophys. Res.* ***106****, 23073-23095; DOI: 10.1029/2001JD000807 (2001).*

25. Park, R. J., Jacob, D. J., Kumar, N. & Yantosca, R. M. Regional visibility statistics in the United States: Natural and transboundary pollution influences, and implications for the Regional Haze Rule. *Atmos. Environ.* ***40****, 5405-5423; DOI: 10.1016/j.atmosenv.2006.04.059 (2006).*

26. Park, R. J., Jacob, D. J., Field, B. D., Yantosca, R. M. & Chin, M. Natural and transboundary pollution influences on sulphate-nitrate-ammonium aerosols in the United States: implications for policy. *J. Geophys. Res.* ***109****, D15204; DOI: 10.1029/2003JD004473 (2004).*

27. Fountoukis, C. & Nenes, A. ISORROPIAII: A computationally efficient thermodynamic equilibrium model for K^+^-Ca^2+^-Mg^2+^-NH_4_ ^+^-Na^+^-SO_4_ ^2-^-NO_3_ --Cl^-^-H_2_O aerosols. *Atmos. Chem. Phys.* ***7****, 4639-4659; DOI: 10.5194/acp-7-4639-2007 (2007).*

28. Streets, D. G. *et al.* An inventory of gaseous and primary aerosol emissions in Asia in the year 2000. *J. Geophys. Res.* ***108****, 8809; DOI: 10.1029/2002JD003093 (2003).*

29. Ohara, T. *et al.* An Asian emission inventory of anthropogenic emission sources for the period 1980-2020. *Atmos. Chem. Phys.* ***7****, 4419-4444; DOI: 10.5194/acp-7-4419-2007 (2007).*

30. Neale, R. *et al.* *NCAR Tech. Note NCAR/TN-486+STR (NCAR,Boulder CO, 2010).*

31. Rayner, N. A. *et al.* Global analyses of sea surface temperature, sea ice, and night marine air temperature since the late nineteenth century. *J. Geophys. Res.* ***108****, 4407; DOI: 10.1029/2002JD002670 (2003).*

32. Liu, X. *et al.* Toward a minimal representation of aerosols in climate models: description and evaluation in the Community Atmosphere Model CAM5. *Geosci. Model Dev.* ***5****, 709-739; DOI: 10.5194/gmd-5-709-2012 (2012).*

33. Lamarque, J. F. *et al.* Historical (1850-2000) gridded anthropogenic and biomass burning emissions of reactive gases and aerosols: Methodology and application. *Atmos. Chem. Phys.* ***10****, 7017-7039; DOI: 10.5194/acp-10-7017-2010 (2010).*

34. IPCC Climate Change 2007: Impacts, Adaptations and Vunerability (eds Parry, M. L. *et al.*) *(Cambridge Univ. Press, UK, 2007).*

35. Li, J., & Zeng, Q. A unified monsoon index, *Geophy. Res. Letts. 29*(8), *115-111-115-114*, *DOI: 10.1029/2001GL013874 (2002)*.


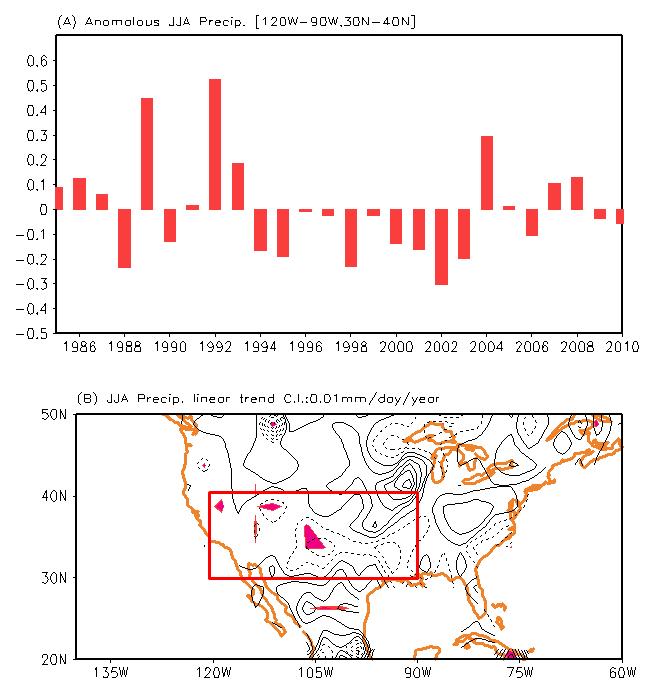


**Supplementary Figure S1** (A) The time series of anomalous precipitation in the western-to-central United States (120°W-90°W, 30°N-40°N) for the period of 1985-2010 during summer (June-July-August). Unit is mm/day. (B) The linear trend of CMAP precipitation for the period of 1985-2010. Contour interval is 0.01mm/day/year and dashed line denotes below zero. Shading in (B) denotes the statistical significance above the 90% confidence level. Box in Supplementary Fig. S1B indicates the region of the western-to-central US (120°W-90°W, 30°N-40°N). GrADS ([http://cola.iges.org](http://cola.iges.org/)) was used to create the map and plot in Fig. S1.


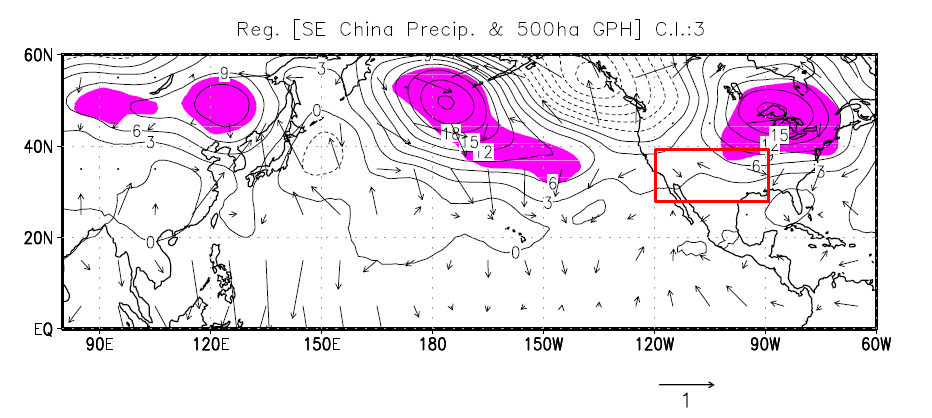


**Supplementary Figure S2** The regressed geopotential height anomalies at 500 hPa from MERRA against the time series of observed precipitation averaged over southeastern China (105°E-120°E, 20°N-35°N). Shading in (A) and (B) denotes the statistical significance above the 90% confidence level. Contour interval is 3 meter/mm/day. The vectors indicate the regressed winds at 850hPa and box indicates the region of western-to-central US (120°W-90°W, 30°N-40°N). Note that the trends in all variables are removed before calculating the regression coefficients. GrADS ([http://cola.iges.org](http://cola.iges.org/)) was used to create the map in Fig. S2.

**Supplementary Figure S3** (A) The differences of zonal (105°E-120°E) averaged temperature differences between the two periods (2001-2010 minus 1985-1994). (B) is the same as in Supplementary Fig. S3A except the wind (shading), and meridional circulation (vector = v; omega × -30). Solid line in Supplementary Fig. S3B indicates the climatological (1985-2010) zonal wind. (C) is the same as in Supplementary Fig. S3A except the divergence field. Units in (A), (B) and (C) are C, m/s and s^-1^, respectively. GrADS ([http://cola.iges.org](http://cola.iges.org/)) was used to create the plots in Fig. S3.

**Supplementary Figure S4** The regressed precipitation map. The regressed precipitation against with the time series of sulphate aerosol averaged in southeastern China (105°E-120°E, 20°N-35°N) for 1985-2010. Shading denotes the region where the statistical significance above the 90% confidence level. Contour interval is 0.2mm/day/μg/m3. GrADS ([http://cola.iges.org](http://cola.iges.org/)) was used to create the map in Fig. S4.

**Supplementary Figure S5** Time series of the concentration of simulated SO_4_^2-^ in SO_2_-run. Simulated SO_4_^2-^ concentrations averaged over southeastern China (105°E-120°E and 20°N-35°N). Unit is μg/m3. GrADS ([http://cola.iges.org](http://cola.iges.org/)) was used to create the plot in Fig. S5.

**Supplementary Figure S6** (a) Regressed precipitation against the EASM index from the CMAP. (b) Same as (a) but for the CAM5 SST-SO_2_ run. Unit is mm day^-1^. Dotted denoted the statistical significance at the 95% confidence level. GrADS ([http://cola.iges.org](http://cola.iges.org/)) was used to create the maps in Fig. S6.

**Supplementary Figure S7** Time-series of the EASM index from (a) the NCEP DOE RA2, (b) the CAM5 SST-SO_2_ run. GrADS ([http://cola.iges.org](http://cola.iges.org/)) was used to create the plots in Fig. S7.

**Supplementary Figure S8** The difference of summer precipitation simulated in the SST-SO_2_ run between the two periods (i.e., 2001-2010 minus 1985-94). Unit is mm/day/year and Contour interval is 0.1 mm/day/year. GrADS ([http://cola.iges.org](http://cola.iges.org/)) was used to create the map in Fig. S8.

**Supplementary Figure S9** (A) The differences of zonal (105°E-120°E) averaged temperature differences between the two periods (2001-2010 minus 1985-1994) in the SO_2_-run. (B) is the same as in Supplementary Fig. S7A except the wind (shading), and meridional circulation (vector = v; omega × -30). Solid line in Supplementary Fig. S7B indicates the climatological (1985-2010) zonal wind in the SO_2_-run. Units in (A) and (B) are °C and m/s, respectively. GrADS ([http://cola.iges.org](http://cola.iges.org/)) was used to create the plots in Fig. S9.


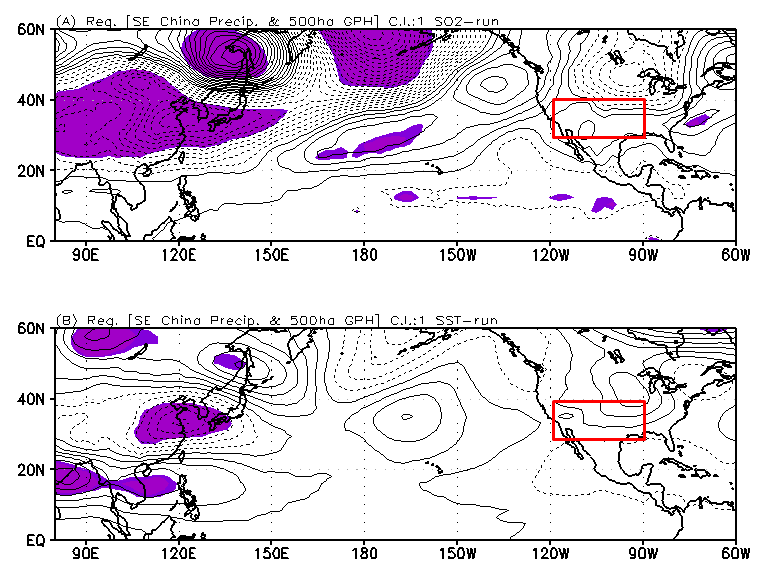


**Supplementary Figure S10** (A) Regressed geopotential height anomalies simulated in the SO_2_-run at 500 hPa against the time series of simulated precipitation averaged over southeastern China. Contour interval is 1 meter/mm/day and shading denotes the statistical significance above the 90% confidence level. (B) is the same as in Supplementary Fig. S8A except the SST-run. Box in (A) and (B) indicates the region of western-to-central US (120°W-90°W, 30°N-40°N). GrADS ([http://cola.iges.org](http://cola.iges.org/)) was used to create the maps in Fig. S10.

**Supplementary Figure S11** The difference of prescribed SST during summer between the two periods (i.e., 2001-2010 minus 1985-1994) in the SST run. Contour interval is 0.2°C. GrADS ([http://cola.iges.org](http://cola.iges.org/)) was used to create the map in Fig. S11.


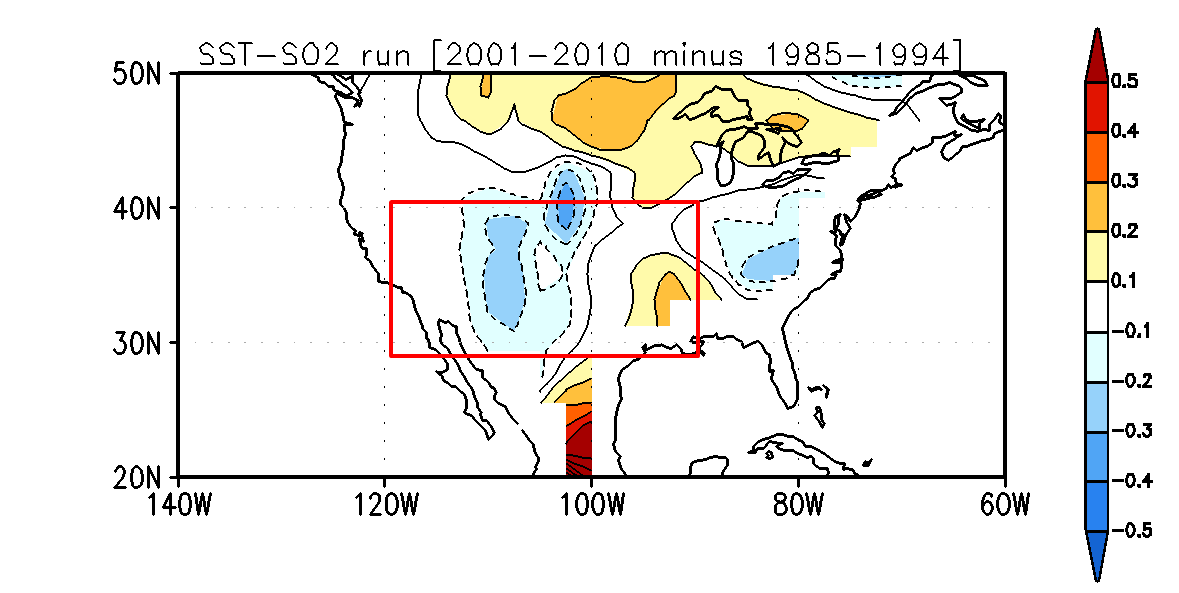


**Supplementary Figure S12** The difference of precipitation simulated in the SST-SO_2_ run in the United States during summer between the two periods (i.e., 2001-2010 minus 1985-94). Unit is mm/day. Box indicates the region of the western-to-central US (120°W-90°W, 30°N-40°N). GrADS ([http://cola.iges.org](http://cola.iges.org/)) was used to create the map in Fig. S12.
